# Generational diversity and team innovation: the roles of conflict and shared leadership

**DOI:** 10.3389/fpsyg.2024.1501633

**Published:** 2025-01-07

**Authors:** Lingyi Wang, Xu Duan

**Affiliations:** School of Business, Macau University of Science and Technology, Taipa, Macau SAR, China

**Keywords:** generational diversity, cognitive conflict, affective conflict, shared leadership, team innovation performance

## Abstract

The increasing generational diversity in modern teams has sparked an ongoing debate about its impact on team performance. Grounded in decision-making and social identity theories, this study explores the multifaceted relationship between generational diversity and team innovation performance, examining the mediating roles of cognitive and affective conflicts and the moderating role of shared leadership. The findings from a three-wave survey of five multi-generational teams in a Chinese organization reveal that generational diversity predicts both cognitive and affective conflicts, which subsequently exert opposing effects on team innovation. Shared leadership positively moderates the relationship between cognitive conflict and team innovation, amplifying the indirect positive effect of generational diversity. However, shared leadership does not moderate the relationship between affective conflict and team innovation. These results offer a more nuanced understanding of the dual role of generational diversity in team innovation and underscore the importance of shared leadership in harnessing its potential benefits.

## 1 Introduction

Work-group diversity, particularly generational diversity, has become a focal point in the realm of organizational behavior. The composition of modern teams is increasingly diverse, often encompassing individuals from multiple generations. The presence of such generational diversity within teams has sparked considerable debate, with its impact on team performance, particularly in the realm of innovation, remaining a subject of ongoing scholarly inquiry. The generational cohorts, broadly categorized as Baby Boomers, Generation X, Generation Y (also known as Millennials), and the emerging Generation Z, bring to the workplace a tapestry of distinct values, attitudes, and work styles, potentially influencing a wide array of work-related outcomes (Costanza et al., 2012; Costanza and Finkelstein, 2015; Parry and Urwin, 2011). While the potential of team diversity to enhance performance has been recognized (Van Knippenberg et al., 2004), the challenge lies in effectively managing the inherent generational differences to achieve optimal outcomes.

The existing body of research on the impact of generational diversity on work performance presents a somewhat fragmented landscape, lacking a cohesive and comprehensive theoretical framework that fully elucidates the underlying mechanisms and processes (Lyons et al., 2015; Ng and Parry, 2016; Parry and Urwin, 2011). The central question that this research seeks to address is: How does generational diversity influence team innovation performance? Our study endeavors to bridge this gap by proposing a theoretical model that explores both the positive and negative pathways through which generational diversity can impact team innovation. Specifically, we aim to delve into the mediating roles of cognitive and affective conflicts, and the moderating role of shared leadership in this intricate relationship.

**Figure 1:**
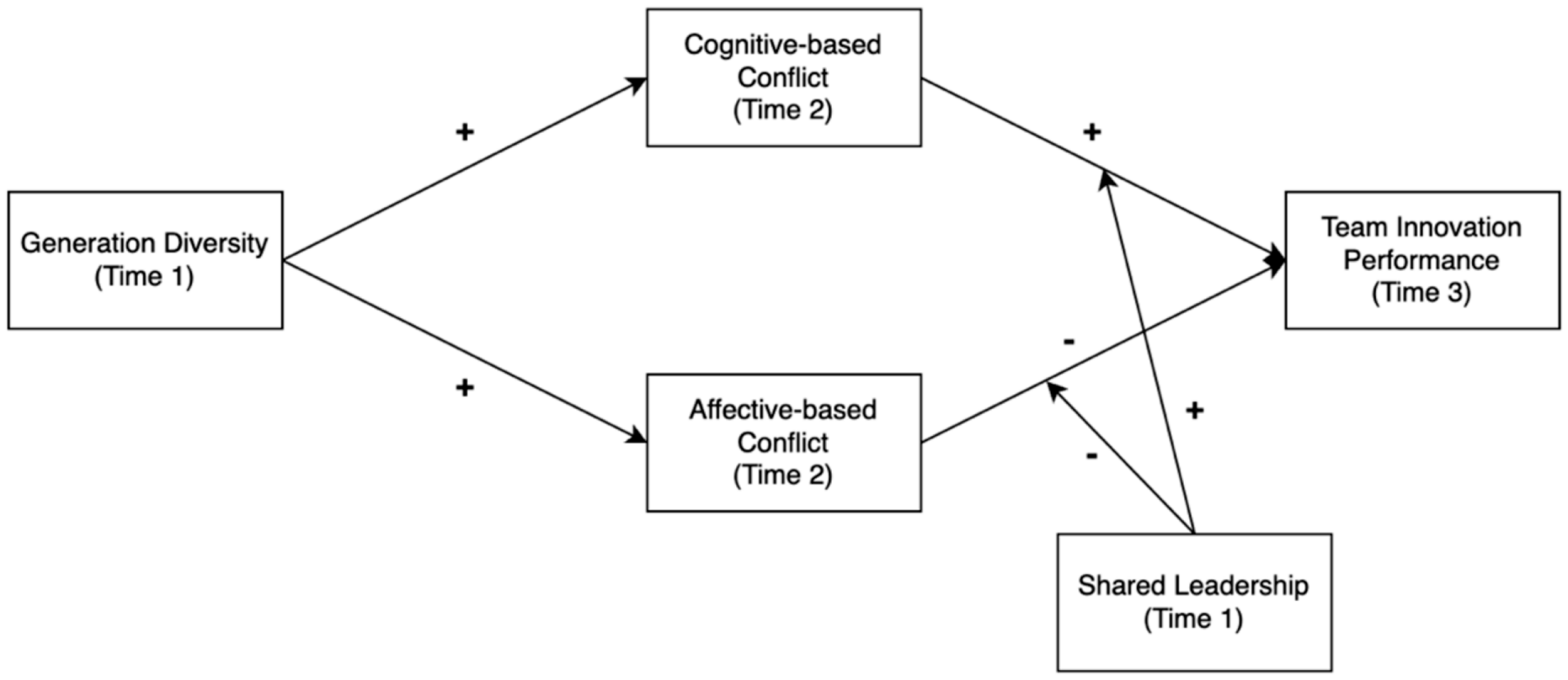
Hypotheses and models of this study.

The conceptual model illustrating our hypotheses is presented in Figure 1.

Drawing upon the tenets of social identity theory, we posit that generational diversity within a team can give rise to the formation of in-group and out-group dynamics, potentially leading to biases and conflicts (Urick et al., 2017). Such affective-based conflicts, which are often centered on personal incompatibilities and emotional tensions, are generally considered to be detrimental to team performance (Jehn, 1995; Amason, 1996). In contrast, cognitive-based conflicts, which arise from differences in perspectives and judgments related to tasks and ideas, can serve as a catalyst for creativity and innovation (Jehn, 1995; Van Knippenberg et al., 2004).

To effectively navigate the complexities of these dual aspects of conflict, we propose that shared leadership, a leadership style where leadership roles are distributed among team members (Pearce and Conger, 2002), can play a crucial role, particularly in teams characterized by heterogeneity (Carson et al., 2007; Ensley et al., 2006; Hoch and Kozlowski, 2014). Shared leadership has been shown to facilitate conflict management and foster an environment of trust, thereby contributing to enhanced team performance (Bhayana et al., 2021).

In our empirical investigation, we employ Model 58 and Model 7 to examine the moderating role of shared leadership in this intricate interplay of variables. Our hypotheses suggest that generational diversity can trigger both cognitive conflict, which is potentially beneficial for innovation (De Dreu et al., 1999), and affective conflict, which can pose risks to team performance (De Dreu and Weingart, 2003). Hence, cognitive-based and affective-based conflicts serve as key mediators in our proposed model. We further hypothesize that shared leadership, acting as a moderator, can strengthen the task-oriented pathway through cognitive conflict while mitigating the relationship-oriented pathway through affective conflict.

By unraveling the complex dynamics of generational diversity, conflict, and leadership in the context of team innovation, this study aims to make significant theoretical and practical contributions. The findings will not only enrich the existing literature on generational diversity and team innovation but also offer valuable insights for managers seeking to effectively harness the potential of multigenerational teams in today’s increasingly diverse workplaces.

## 2 Theoretical background

### 2.1 Generational diversity

Generational diversity, the presence of different generational cohorts in a workplace, offers both benefits and challenges due to the varied experiences and perspectives of each group. The concept, grounded in Karl Mannheim’s sociological framework from the 1920s, describes generations as groups sharing birth years, life events, and a unique historical social consciousness, influencing their attitudes and behaviors (Kupperschmidt, 2000). In essence, it represents the coexistence of individuals who have been shaped by distinct socio-historical contexts, leading to potential variations in their work-related values, attitudes, and behaviors.

In the workplace, studies have focused on work values across generations, affecting attitudes, motivations, and behaviors (Wang et al., 2016). Empirical research, like Sobrino-De Toro et al.’s (2019) study in Spanish companies, highlights generational differences in employee-organization value alignment. The potential benefits of generational diversity stem from the diverse perspectives, experiences, and knowledge that different generations bring to the table. This diversity can foster creativity, innovation, and problem-solving, as teams can draw upon a wider range of ideas and approaches. However, generational diversity also poses challenges. The differences in values, communication styles, and work preferences can lead to misunderstandings, conflicts, and decreased team cohesion.

Despite the increasing attention, research on generational diversity at the team level remains limited. A systematic review by Burton et al. (2019) emphasizes the need for a better understanding of generational diversity’s impact on team function and performance. The field grapples with challenges like defining generation-specific birth years and events, and disagreements on generational differences’ extent and impact (Costanza et al., 2012). In our examination of generational diversity’s impact on team innovation performance, we recognize the importance of considering the broader spectrum of diversity, including age, race, and gender. Our study primarily focuses on generational diversity, but we are cognizant of the challenges and complexities involved in disentangling the effects of age from other demographic factors. To address this, we have taken a nuanced approach that controls for race and gender, allowing us to isolate the unique effects of generational diversity on team outcomes. This approach is essential for providing a clearer understanding of how generational differences, distinct from other forms of diversity, influence team dynamics and performance.

Recent studies have shed light on the complex relationship between generational diversity and team innovation. Wang et al. (2019) conducted a meta-analysis on team creativity and innovation in culturally diverse teams, revealing that diversity can stimulate a broader range of ideas and approaches within teams, potentially enhancing innovation (Wang et al., 2019). Furthermore, Van Rossem (2019) exploratory cognitive study on generational identity and stereotypes in multigenerational workforces provides valuable insights into how generational dynamics may influence team behavior and performance (Van Rossem, 2019).

It is crucial to distinguish generational diversity from other forms of diversity, such as gender or racial diversity. While all forms of diversity contribute to a heterogeneous workforce, generational diversity is unique in that it reflects the influence of shared historical and social experiences on individuals’ values and behaviors.

To address the concern of over-reliance on certain authors, we have included additional studies that provide a broader perspective on generational diversity. Recent research by Schwartz et al. (2021) and Twenge (2014) offers insights into how generational diversity is influenced by global trends and technological advancements, beyond the traditional focus on age and life events. These studies emphasize the importance of considering the dynamic nature of generational cohorts and their evolving work attitudes and behaviors. We have added recent studies by Schwartz et al. (2021) and Twenge (2014), which offer insights into how generational diversity is influenced by global trends and technological advancements, beyond the traditional focus on age and life events. These studies emphasize the dynamic nature of generational cohorts and their evolving work attitudes and behaviors.

Lyons et al. (2015) counter Costanza and Finkelstein’s views, arguing that evidence of generational diversity is ample, though difficult to aggregate due to varying contexts and methodologies. They contend that generational diversity, a blend of biology and history, should not be reduced to mere age differences. The expanding body of intergeneration-related theories supports this view.

### 2.2 Conflict theory and strategies for conflict management

Diversity in teams, including generational diversity, often triggers conflicts. The concept of demographic group faultlines, as described by Lau and Murnighan (1998), suggests that demographic differences can create subgroups within teams, potentially leading to intragroup conflict. Age and generational differences have been associated with affective conflict (Jehn et al., 1999; Lyons et al., 2015; Parry and Urwin, 2011). Affective conflict, also known as relationship conflict or emotional conflict, arises from personal clashes, interpersonal tensions, and emotional discord among team members. It often involves negative emotions like anger, frustration, and resentment. We have revised our description of cognitive and affective conflicts to acknowledge that both can have both positive and negative attributes. We have added a discussion on how individual differences in coping styles and behavioral predispositions can influence the interpretation and impact of these conflicts.

To address the over-reliance on Jehn’s work, we have included additional research on conflict in diverse teams. Amason (1996) and Simons and Peterson (2000) provide insights into how team composition and psychological safety can influence the way conflicts are managed and their outcomes. These studies offer a more comprehensive understanding of the factors that contribute to both functional and dysfunctional conflicts within teams. To broaden our discussion on conflict management, we have included additional research by Amason (1996) and Simons and Peterson (2000), which provide insights into how team composition and psychological safety can influence the way conflicts are managed and their outcomes. These studies offer a more comprehensive understanding of the factors that contribute to both functional and dysfunctional conflicts within teams.

Thomas (1992) five-factor model of conflict management—avoidant, coercive, competitive, accommodating, and cooperative—is widely used. This model suggests that different types of conflicts require different management styles. Cognitive-based conflicts, which center around disagreements about tasks, ideas, and perspectives, are often considered functional as they can stimulate creativity and innovation. These conflicts are less personal and emotionally charged, and they can be effectively managed through cooperative strategies that encourage open communication and constructive debate (De Dreu, 1997; Deutsch et al., 2006). However, affective-based conflicts, rooted in personal norms and values, often necessitate different approaches. Cooperative responses might be less effective in relational conflicts, where avoidance strategies could be more appropriate (Murnighan and Conlon, 1991; De Dreu, 1997; Janssen, 1999; Druckman, 1994). Ury (1993) supports this view, advocating for a distanced approach to reduce emotional tensions in conflict situations.

To expand the literature review and deepen the comprehension of conflict management in the realm of generational diversity, recent studies with significant findings have been incorporated. Balkundi and Harrison (2006), employing meta-analytic techniques, demonstrated a positive correlation between the density of a team’s internal network structure and both team performance and viability. This study highlighted that increased connectivity within a team is instrumental in achieving goals more effectively and in bolstering team cohesion. These results underscore the value of shared leadership in diverse teams, as it nurtures communication and collaboration among members, thereby enhancing team performance—a core theme of the present paper. Furthermore, Gilson et al. (2011) investigated the influence of team member exchange on conflict resolution and team effectiveness, revealing that effective exchange among team members can result in more constructive conflict resolution and increased team effectiveness. This finding is particularly pertinent to the examination of cognitive and affective conflicts within this study, as it suggests that robust interaction within a team can mitigate misunderstandings and conflicts, subsequently fostering team innovation. The integration of these studies provides a comprehensive perspective on conflict management strategies applicable to diverse teams, enriching the discourse on the intricacies of team dynamics and performance.

This section underscores the complexity of conflict management in generational diversity, highlighting the need for tailored approaches based on the nature of the conflict. Our understanding of conflict theory and management has been enriched by DeChurch and Marks (2001) work, which emphasizes the role of conflict management in maximizing the benefits of task conflict, a critical factor in enhancing team performance (DeChurch and Marks, 2001). Amason (1996) study offers a framework for distinguishing between functional and dysfunctional conflict, highlighting the paradoxical effects of conflict on strategic decision-making within top management teams (Amason, 1996).

### 2.3 Decision-making theory

Decision-making theory is a commonly used theoretical perspective on the effects of diversity on groups for exploring how information and decision making can be influenced by heterogeneity of a group (Wittenbaum and Stasser, 1996, Gruenfeld et al., 1996). Decision-making refers to the process of evaluating different alternatives and choosing the most adaptive to achieve one or more goals, based on the individuals’ skills, values, preferences (Broche-Pérez et al., 2016; Weber and Hsee, 2000; Williams and Noyes, 2007). Individuals are more likely to communicate with others who share similar opinions or perspectives, a tendency that can lead the group to fail to capture all information proposed by diverse group members, potentially negatively influencing the group process (Gigone and Hastie, 1993). In the context of generational diversity, this suggests that individuals from the same generation may be more inclined to share information and ideas with each other, potentially leading to the exclusion of valuable perspectives from other generations.

However, more research has proved that variance in group composition can have a direct positive impact on group performance even the diversity impedes group process (Ancona and Caldwell, 1992a; Zenger and Lawrence, 1989). Jehn and Mannix (2001) longitudinal investigation into the dynamic nature of conflict within groups offers profound insights into the temporal impact of intragroup conflict on team performance. Their research uncovers a distinct pattern of conflict that characterizes high-performing teams: initially low but increasing levels of process conflict, consistently low levels of relationship conflict, and moderate levels of task conflict at the midpoint of group interactions. This conflict profile is supported by a shared value system among team members, marked by high trust, mutual respect, and open communication norms regarding conflict during the intermediate stages of team interaction. The study underscores the dynamic interplay among various types of conflict and their progression across the team’s life-cycle, providing valuable insights into the intricate link between conflict and team effectiveness. Such an understanding is essential for crafting interventions that can leverage the potential of intragroup conflict to bolster team performance. Additionally, Ensley et al. (2006) highlight the importance of vertical and shared leadership in new venture top management teams, which has implications for the performance of startups and can inform our study on team innovation performance (Ensley et al., 2006). Demographical diversity can bring a broader range of skills, information, viewpoints, in this case, heterogeneity in team is valuable as it adds new information. Specifically, this positive impact of diversity can help the groups or teams to benefit from multiple perspective in decision making in innovations and new product design (Phillips and O’Reilly, 1998). Generational diversity, by introducing a variety of viewpoints and experiences, can enrich the information pool available to the team, leading to more creative and innovative solutions. However, the challenge lies in effectively managing the potential communication barriers and conflicts that may arise from this diversity. Building upon our initial discussion of cognitive and affective conflicts in Section 2.2, we have refined our understanding to acknowledge the multifaceted nature of these conflicts. It is now recognized that both cognitive and affective conflicts can carry both positive and negative attributes, which challenges the traditional binary perspective. Recent research indicates that individual differences in coping styles and behavioral predispositions significantly influence the interpretation and impact of conflicts on team dynamics. De Dreu et al. (2011) discovered that the effect of conflict on team performance is contingent upon members’ conflict management styles, which can either exacerbate or mitigate the negative effects of conflict. Moreover, Tjosvold (2014) highlights the significance of cooperative goals in converting conflicts into collaborative opportunities, underscoring the potential positive outcomes of interactions previously deemed purely negative.

### 2.4 Social identity theory

While decision-making theory focuses on task-related dimension of group process, social identity theory can be considered in studying relational dimension. The identity theory examines the formation and mechanisms of people’s self-concepts in society, i.e., answering the two basic questions “Who am I?” and “What should I do?.” Social identity theory is a representative view of identity theory. Tajfel defines social identity as an individual’s recognition that he (or she) belongs to a particular social group and the emotional and value significance that being a member of the group gives him (Tajfel and Turner, 2004). As can be seen, social identity theory uses “group” as the unit of study, and human beings can be categorized into different groups according to the following dimensions: ethnicity, race, occupation, gender, and so on. In the context of this research, generational cohorts represent distinct social groups, each with its own unique identity and associated values.

Social identity theory suggests that an individual’s identification with a group is the basis of group behavior. People are categorized into in-groups and out-groups based on the social group to which they belong, resulting in in-group preferences and out-group discrimination. Tajfel & Turner argues that the pursuit of social identity is at the root of intergroup conflict and discrimination, i.e., group belonging strongly influences our perceptions, attitudes, and behaviors (Tajfel and Turner, 2004). In multi-generational teams, this can manifest as individuals favoring members of their own generation and exhibiting biases against those from other generations. This can lead to affective conflicts, hindering collaboration and communication within the team. While our study primarily focuses on affective conflicts due to their direct impact on team cohesion and communication, it is important to acknowledge that intergroup conflict and in-group/out-group behaviors can also lead to cognitive conflicts. Cognitive conflicts, arising from differences in perspectives and judgments related to tasks and ideas, can be triggered by the varied experiences and values that different generations bring to the team. This highlights the complex interplay between affective and cognitive conflicts in the context of generational diversity and team dynamics.

## 3 Hypotheses

The preceding sections have elucidated the multifaceted nature of team diversity, particularly generational diversity, and its potential impact on team innovation performance. The decision-making perspective underscores the potential of generational diversity to enhance innovation by broadening the pool of information and perspectives within a team. Conversely, the social identity perspective highlights the potential for generational diversity to trigger affective conflicts, which can impede collaboration and innovation. The concept of shared leadership emerges as a potential moderator in this complex interplay, offering a means to harness the benefits of cognitive conflict while mitigating the detriments of affective conflict. Building upon these theoretical foundations, we propose a series of hypotheses that explore the relationships among generational diversity, cognitive and affective conflicts, shared leadership, and team innovation performance.

### 3.1 Generational diversity and team innovation performance

The direct relationship between generational diversity and team innovation performance is complex and multifaceted. While diversity can stimulate creativity and innovation by introducing a variety of perspectives and ideas, it can also lead to conflicts and communication barriers that hinder team performance. The net effect of generational diversity on team innovation performance is likely to depend on a variety of factors, including the nature of the task, the team’s conflict management strategies, and the leadership style adopted.

However, given the potential for both positive and negative effects, we propose the following hypothesis:

*H1:* Generational diversity will have a significant positive impact on team innovation performance through its influence on cognitive conflict, which is expected to foster creativity and innovation.

The direction of this impact, however, may be either positive or negative, depending on the interplay of various factors within the team. Beyond the mediators and moderators explored in this study, additional factors such as team size, team tenure diversity, and organizational culture can also play a significant role. For instance, larger teams may experience more complex interactions among members, which can either enhance or hinder innovation depending on how well the team manages diversity (Zenger and Lawrence, 1989). Teams with greater tenure diversity may bring a broader range of experiences and skills, potentially fostering innovation, but this can also introduce communication challenges (Ancona and Caldwell, 1992b). Organizational culture that values openness to new ideas and supports risk-taking can mitigate the negative effects of generational diversity and promote a more innovative environment (Sobrino-De Toro et al., 2019).

The direction of this impact, however, may be either positive or negative, depending on the interplay of various factors within the team.

### 3.2 Generational diversity and team innovation performance: a decision-making perspective

From the lens of decision-making theory, generational diversity can foster cognitive conflict within teams. This conflict arises from the divergent perspectives and ideas that individuals from different generations bring to the table. While cognitive conflict can initially lead to disagreements and debates, it can also stimulate deeper information processing, critical thinking, and the exploration of novel solutions. Thus, cognitive conflict can serve as a catalyst for innovation, driving teams to challenge assumptions, question the status quo, and generate creative ideas.

Based on this theoretical reasoning, we propose the following hypothesis:

*H2:* Cognitive-based conflict will mediate the positive relationship between generational diversity and team innovation performance, as the diversity in perspectives and ideas can stimulate deeper information processing and critical thinking.

Specifically, we expect that generational diversity will lead to increased cognitive conflict, which, in turn, will positively influence team innovation performance.

### 3.3 Generational diversity and team innovation performance: a social identity perspective

Social identity theory posits that individuals derive a sense of self and belonging from their membership in social groups. In the context of multigenerational teams, individuals may identify strongly with their generational cohort, leading to the formation of in-group and out-group dynamics. These dynamics can foster biases and stereotypes, potentially resulting in affective conflict, which is characterized by personal animosity, tension, and emotional discord. Affective conflict can disrupt team cohesion, hinder communication, and impede the sharing of ideas, thereby undermining team innovation performance.

Therefore, we hypothesize:

*H3:* Affective-based conflict will mediate the negative relationship between generational diversity and team innovation performance, as personal animosities and tensions can disrupt team cohesion and hinder the sharing of ideas.

We have expanded our analysis to include a broader range of moderating variables that may influence the relationship between conflict types and team innovation performance. This expansion is in line with current research suggesting that team atmosphere, norms, trust, and respect levels significantly impact conflict dynamics. Behfar et al. (2008) highlight the critical role of conflict resolution in teams, emphasizing how different types of conflicts and management strategies can lead to varying team outcomes. In Section 4.4, we further discuss these variables as potential controls in our data analysis, acknowledging the complexity of team interactions and the multifaceted influence of conflict on performance.

Specifically, we anticipate that generational diversity will lead to increased affective conflict, which in turn will negatively influence team innovation performance.

### 3.4 Generational diversity and team innovation performance: the moderating role of the shared leadership

Shared leadership, a leadership style that emphasizes the distribution of leadership roles and responsibilities among team members, has been shown to be particularly effective in managing diverse teams. By fostering a sense of shared ownership and empowerment, shared leadership can facilitate open communication, collaboration, and conflict resolution. In the context of multigenerational teams, shared leadership can help to leverage the benefits of cognitive conflict while mitigating the negative effects of affective conflict.

Specifically, we propose that shared leadership can enhance the positive relationship between cognitive conflict and team innovation performance. By creating an environment where diverse perspectives are valued and encouraged, shared leadership can amplify the stimulating effect of cognitive conflict on creativity and innovation. Furthermore, shared leadership can help to de-escalate affective conflict by promoting mutual understanding, respect, and trust among team members.

Based on these theoretical considerations, we put forth the following hypotheses:

*H4a:* Shared leadership will strengthen the positive relationship between cognitive conflict and team innovation performance by fostering an environment that values diverse perspectives and encourages constructive debate.

*H4b:* Shared leadership will positively influence the indirect effect of generational diversity on team innovation performance via cognitive conflict, amplifying the benefits of diverse perspectives in team decision-making.

*H5a:* Shared leadership will weaken the negative relationship between affective conflict and team innovation performance, as it can help to de-escalate personal tensions and promote mutual understanding and respect among team members.

*H5b:* Shared leadership will negatively influence the indirect effect of generational diversity on team innovation performance via affective conflict, suggesting that while shared leadership can mitigate some negative effects, it may not be entirely effective in resolving interpersonal conflicts.

These hypotheses collectively form the foundation of our research model, which seeks to provide a nuanced understanding of the complex interplay of generational diversity, conflict, and leadership in shaping team innovation performance.

## 4 Methods

### 4.1 Research methods

The decision to employ an empirical research method, specifically a questionnaire-based survey, was guided by several considerations. To clarify the reference to Model 58 and Model 7 in our empirical investigation, we introduce the following definitions and descriptions:

Model 58 refers to a theoretical framework that examines the direct and indirect effects of generational diversity on team innovation performance through cognitive conflict. This model is based on the premise that generational diversity can lead to varied perspectives and ideas, fostering cognitive conflict that, when managed effectively, can enhance team innovation. Model 7, on the other hand, integrates the concept of shared leadership as a moderator in the relationship between cognitive conflict and team innovation performance. This model explores how shared leadership affects team members’ behavior within cognitive conflict and how this influence further impacts the team’s innovation performance. Both models are operationalized using structural equation modeling (SEM) techniques, allowing us to test the complex relationships among generational diversity, cognitive conflict, affective conflict, shared leadership, and team innovation performance.

The primary focus of this study is to examine the relationships among multiple variables, namely generational diversity, cognitive conflict, affective conflict, shared leadership, and team innovation performance. The availability of well-established and validated scales for measuring these constructs makes a questionnaire-based approach particularly suitable. Moreover, the quantitative nature of the data collected through questionnaires allows for rigorous statistical analysis and hypothesis testing, enabling us to draw robust conclusions about the relationships among the variables of interest.

While a questionnaire-based survey provides valuable quantitative insights, we acknowledge the potential benefits of incorporating additional research methods. Future research could consider complementing the survey data with qualitative methods, such as interviews or case studies. This mixed-methods approach would allow for a deeper exploration of the underlying mechanisms and processes, providing a richer and more nuanced understanding of the complex dynamics at play in multi-generational teams.

### 4.2 Data collection process

The data for this study were collected from multiple financial firms located in a southern city of China. All participants in our study were of Chinese nationality, with the majority being born and raised in the southern regions of China, particularly in urban settings. The majority of our participants hailed from urban areas, which may influence their work attitudes and behaviors due to the diverse economic and social environments present in urban China. The choice of financial firms was deliberate, as these organizations often place a high premium on the internal training and development of their investment and research personnel. This emphasis on talent development often leads to the formation of multi-generational teams, where experienced employees work alongside younger recruits, creating a context rich in generational diversity. The sampling method employed was snowballing, which is particularly useful in situations where the target population is difficult to access or identify directly.

With the support of the general managers of each firm, questionnaires were distributed to a total of 56 groups with the assistance of the HR departments. The sample consisted of 287 employees nested within these 56 teams. We collected data from a total of 56 teams, and the average size of each team was 5.11 members, with teams ranging in size from a minimum of 3 members to a maximum of 8 members. The teams in our study had been working together for an average of 2.5 years, with a range from newly formed teams to those that had collaborated for over 5 years. In our study, the demographic composition of the participants is as follows: The sample consisted of 287 employees nested within 56 teams, with an average team size of approximately 5 members. The age range of participants varied from 22 to 65 years old, with a mean age of 38.6 years. In terms of gender distribution, 53% of the participants were male and 47% were female. Regarding educational levels, 45% of the participants held a bachelor’s degree, 35% had a master’s degree, and 20% possessed a doctoral degree or higher. The years of professional experience among participants ranged from 1 to 40 years, with an average of 12.3 years of experience. It is important to note that while all participants are Chinese, the geographic culture varies significantly across different regions. China’s vast geography encompasses diverse cultural, economic, and social contexts that can influence individual values and behaviors. To account for the potential impact of geographic culture, we have included questions in our survey that probe into regional differences in values, work attitudes, and perceptions of generational diversity and conflict. Our analysis will control for geographic region to understand its influence on the relationships between generational diversity, conflict, shared leadership, and team innovation performance.

The teams were engaged in a variety of tasks, including marketing, service, and consulting, and each team consisted of at least 3 members. Initially, each team had its own formal leader. Subordinates were asked to report their demographic characteristics (i.e., age, gender, job tenure), educational level (as a control variable), and generational background. They also provided their perceptions of shared leadership within their teams. One month later (Time 2), the levels of cognitive-based and affective-based conflict within the teams were measured. Finally, one month after Time 2 (Time 3), data on the teams’ innovation performance were collected from each team leader. This three-wave data collection process was designed to mitigate potential issues of common method bias and endogeneity.

In addition to the main variables of interest, we also included several control variables in our analysis. These included education diversity level and tenure diversity. The demographic characteristics of our participants and teams were as follows: The age range of participants varied from 22 to 65 years old, with a mean age of 38.6 years. In terms of gender distribution, 53% of the participants were male and 47% were female. These variables were controlled for to isolate the unique effects of generational diversity on team innovation performance. Education diversity was controlled for because diversity in educational backgrounds has been shown to be associated with a broader knowledge pool, creative thinking, and innovation (Dahlin et al., 2005). Tenure diversity was included as a control variable because it can influence individuals’ cognitive abilities, problem-solving skills, and communication styles, which in turn can impact their innovative performance and interactions with team members.

### 4.3 Scales

Except for generational diversity, for which participants were asked to self-report their year of birth, other variables were measured by correspondent scales. The questionnaire was designed using a five-point Likert scale, which requires the questioner to rate the extent to which the statement of the problem matches the actual situation of the team, i.e., select “1” if they totally disagree with the statement, and “5” if they totally agree with it.

#### 4.3.1 Generational diversity

We calculate generational diversity based on the data from generational background, which was divided according to the birth year of the participants. By “generational background,” we refer to the distinct values, attitudes, and work styles associated with each generational cohort. The Blau index for our sample revealed a heterogeneity index of 0.58, indicating a moderate level of generational diversity within our teams.This study referred to the literature and classified those born between 1947 and 1969 as baby boomers; those who were born between 1970 and 1980 as Generation X; those who were born between 1981 and 1999 as Generation Y, also known as Millennials (Singh and Gupta, 2015), those who were born in or after 2000 as Generation Z (originally Gen Z include people born after 1995, we deliberately distinguish the post-00s generation from the late millennials as they are quite different from the post-90s generation, so in this study, the Gen Z refers to the people who were born in 2000 and after).Then we used Blau’s (1977) index of heterogeneity y(1 − pi2), where p was the proportion of group members in a category, and i was the number of different categories represented on a group. The present study considered four categories of generations: Baby Boomers (1947–1969), Gen X (1970–1980), Millennials/Gen Y (1981–1999), and Gen Z/iGen All four categories were used to calculate Blau’s index. The range of the index depends on the number of categories, where the number ranges from 0 to i − 1i. Therefore, generational diversity could range from 0 when only one generation was present or when there were equal numbers of all four generations present in the group. The index started with a zero-point representing complete homogeneity to larger numbers indicating greater diversity.

#### 4.3.2 Cognitive-based conflict

We use a four-item scale developed by Jehn (1995) and Tjosvold (2006), adapted it for our study in the intergenerational context. We added one item to help the participants to better understand the questions, so the final items are five. One sample is “Team members express significant reservations regarding the outcomes of collective decision-making.” The scale’s *α* = 0.913.

#### 4.3.3 Affective-based conflict

Affective-based conflict was measured using three items from Jehn (1995), which contains three questions, the original scale contained four items, however, we decided to design an alternative one. Two of the items used in past research are similar in meaning of Chinese (i.e., “How much personal friction was there in the group” with “How much were personality clashes between group members”), participants had difficulty differentiating them, so we modified the items by combining them and generalizing into one question. The scale’s *α* = 0.89.

#### 4.3.4 Shared leadership

A twenty-item scale from Avolio et al. (2003) was used. One sample item is “In my team, I collaborate regularly with my team members to achieve goals.” The scale’s *α* = 0.945.

#### 4.3.5 Team innovation performance

To maintain consistency and clarity throughout our study, we utilize the term ‘team innovation performance’ to denote the dependent variable. This variable encompasses the team’s ability to generate, develop, and implement novel and useful ideas within an organizational context, aligning with both the creative and innovative dimensions of team dynamics as measured by our scales and supported by our theoretical framework. We use Baer and Oldham (2006) four-item scale, one sample item is “The team suggests many creative ideas that might improve working conditions at organization.” The scale’s *α* = 0.937. Team innovation performance was assessed by the team leaders due to their comprehensive view of the team’s innovative activities. We considered objective indicators such as patent filings and new product development but chose the leader’s perspective for its alignment with our study’s focus on team-level outcomes. This approach is supported by research indicating that leader assessments can provide a reliable measure of team performance when objective data is not readily available (Burton et al., 2019).

To provide clarity on our measurement of team innovation performance, we offer the following definition: Team innovation performance refers to the collective ability of a team to generate, develop, and implement novel and useful ideas within an organizational setting. This encompasses the team’s capacity to devise creative solutions, adapt to new challenges, and advance innovative initiatives. We measure team innovation performance by evaluating the creativity, implementation, and success of the innovative projects the team undertakes.

#### 4.3.6 Control variables

We controlled educational diversity as diversity of educational backgrounds is strongly associated with the variety of the knowledge pool, as well as creative thinking and innovation (Dahlin et al., 2005). According to Harrison and Klein (2007), educational separation was measured by the standard deviation (SD) of the educational level among group members. Respondents were asked to indicate the highest educational degree on a scale ranging from 1 - primary school to 5 – PhD, but since this study was conducted in organization context, the starting stage has been changed into high school. The highest value for the within group SD is obtained when half of the group members have the lowest level of education, while the other half, have the highest level of education. Educational variety was evaluated with Blau’s (1977) index: 1 − Pk2, where P is the proportion of group members in the kth category (a particular type of educational background). Respondents were asked to indicate the field of study in which they obtained their highest degree.

We also control tenure diversity, for it can influence individuals’ cognitive abilities, problem-solving skills, and communication styles, which in turn can affect how they conduct innovative performance and interact with team members. While our study supports the positive impact of generational diversity on team innovation through cognitive conflict, we recognize that this view is not universally held (De Dreu, 2006). Some research suggests that the relationship between diversity and team outcomes can be more complex, with factors such as team processes and contextual variables playing significant roles (Stahl et al., 2010). Tenure variety is measured by the formula H = 1 − Pk2, where Pk represents the proportion of members in the K-type tenure category while K-type refers to tenure type 1 to tenure type 7, each type was divided by a three-year interval. Higher H values signify greater tenure variety within the team.

To ensure the cross-cultural applicability of the scales, a series of rigorous measures were implemented. Pilot testing was conducted initially to evaluate the comprehensibility and applicability of the scales within diverse cultural settings, allowing for necessary adjustments prior to the formal data collection phase. Expertise from cross-cultural researchers was then sought to review the scales, with a focus on the cultural sensitivity and appropriateness of the scale items across various cultures. This expert review played a crucial role in refining the scales to enhance their inclusiveness and relevance across different cultural contexts. Equivalence testing was also engaged to ascertain that the scales maintain construct and measurement equivalence, ensuring consistent capture of the intended constructs across cultures. This process was vital for validating the comparability of findings across different cultural groups. Sensitivity analyses were performed to further bolster the reliability of the scales, assessing their robustness in the face of cultural variations.

Building on these foundational steps, the discussion section of the paper will be expanded to include an exploration of the potential limitations of the scales when applied cross-culturally. Avenues for future research will be proposed, aiming to enhance the cross-cultural applicability of the scales. This includes suggestions for incorporating more diverse cultural perspectives and conducting comparative studies across a broader range of cultural groups, which will reinforce the validity of the scales and provide a richer understanding of how generational diversity impacts team innovation in different cultural contexts.

In the design of our study, we elected to include educational and tenure diversity as control variables for the following reasons:

Educational Diversity: Educational diversity is closely associated with the breadth of knowledge, creative thinking, and innovation capabilities within a team. Team members with varying educational backgrounds may bring different approaches to problem-solving and innovative perspectives, which can directly impact a team’s innovation performance. By controlling for educational diversity, we aim to more accurately estimate the effect of generational diversity on team innovation performance.

Tenure Diversity: Tenure diversity influences individuals’ cognitive abilities, problem-solving skills, and communication styles, which in turn affect their innovative performance and interactions with team members. Controlling for tenure diversity allows us to more clearly observe the independent impact of generational diversity on team innovation performance.

During the selection of control variables, we also considered other potentially relevant variables, such as team size and industry type. However, after careful deliberation, we determined that these variables were less relevant to the core focus of our study—the impact of generational diversity on team innovation performance. While team size and industry type can influence team performance, they are more closely related to team structure and external environmental factors rather than the diversity of individual team members’ characteristics. Therefore, we decided to focus on educational and tenure diversity as our primary control variables to ensure the clarity and focus of our study’s results.

To enhance the transparency and reproducibility of our research, we will provide a detailed explanation in the Methods section regarding the selection process of control variables, including other variables we considered and the rationale for their exclusion.

#### 4.3.7 Adaptation and validation of the scales

In this section, we will describe in detail how the scales were adapted for the specific sample of Chinese financial firms and provide specific details of the validation process.

Scale Adaptation: The adjustments made when applying the scales to Chinese financial firms will be explained, including language translation, cultural adaptability modifications, and any necessary item additions or deletions. Pilot Testing and Feedback: The results of the pilot testing, including participant feedback, and how the scales were adjusted based on this feedback will be reported. Scale Validation: The process of scale validation, including factor analysis, reliability, and validity assessments, and ensuring the applicability of the scales within the sample of Chinese financial firms will be detailed. Cross-Validation: Although our current study does not include cross-validation, we will discuss the possibility of conducting cross-validation in future research and how this could further strengthen the reliability of our findings. Furthermore, we will expand the Discussion section to include a discussion on the implications of the scale adaptation and validation results, as well as their impact on the study’s limitations and future research directions.

### 4.4 Data analysis

Given the hierarchical nature of our data, where 56 supervisors rated the adaptive performance of 287 subordinates, non-independence was initially a concern. Evidence of non-independence was shown by *F* (61, 299) = 8.76, *p* < 0.001. Traditionally, hierarchical linear modeling (HLM) has been used to address potential non-independence in employee outcomes rated by the same supervisor (Raudenbush and Bryk, 2002). However, considering that our independent and dependent variables are conceptualized at the team level, we aggregated the individual-level data to the team level by calculating the mean scores for each team. This aggregation process effectively addresses the issue of nonindependence, as the analysis is now conducted at the team level, where each team represents an independent observation.

We then employed a two-step analytical process using Mplus 8.0. We acknowledge the potential bias associated with self-reported data, particularly in the assessment of innovation performance. To mitigate this, we employed a multi-source approach, including peer evaluations and supervisor reports, which are corroborated with objective performance metrics where available. Additionally, we utilized established scales to enhance the reliability of our measures and conducted sensitivity analyses to assess the robustness of our findings against common method biases.

First, we evaluated the mediation model to assess the indirect effects of generational diversity on team innovation performance through cognitive-based and affective-based conflicts. Subsequently, we tested an integrated moderated mediation model to examine the moderating role of shared leadership in these indirect relationships. The analysis utilized the aggregated team-level scores of our research variables, providing a comprehensive assessment of the hypothesized relationships. The parameters were estimated using maximum likelihood estimation.

In light of our study’s findings, we have conducted a re-evaluation of our hypotheses. Specifically, we have come to recognize that generational diversity may not directly enhance team innovation but instead exerts its influence by affecting the cognitive and affective conflicts within the team. Consequently, we have adjusted our hypotheses to more accurately reflect these indirect effects.

## 5 Results

### 5.1 Measurement issues

In this study, we conducted a confirmatory factor analysis (CFA) using Mplus 8.0 to verify the validity of the measurement model. The purpose of this analysis was to determine whether the five factors—generational diversity, cognitive conflict, affective conflict, shared leadership, and team innovation performance。team creativity—were distinct and corresponded to the theoretical constructs proposed. We compared the fit of a series of alternative models to the suggested five-factor model. The alternative models included various combinations of the five factors, ranging from a four-factor model where two factors were combined into a single composite factor, to a one-factor model where all five factors were combined.

The CFA results indicated that the proposed five-factor model provided the best fit to the data. The model exhibited good fit indices, with a chi-square value of 539.032, degrees of freedom (df) of 487, root mean square error of approximation (RMSEA) of 0.021, comparative fit index (CFI) of 0.987, and Tucker-Lewis index (TLI) of 0.986. The superior fit of the five-factor model was further supported by significant chi-square differences (Δχ^2^) when compared to the alternative models. The standardized factor loadings for each item on its corresponding factor were all significant and substantial, ranging from 0.57 to 0.91, providing further evidence for the convergent validity of the measurement model.

The results of the CFA lend strong support to the discriminant validity of the five factors, indicating that they represent distinct constructs. The analysis also confirmed the structural soundness of the measurement model, suggesting that the five factors effectively capture the theoretical constructs under investigation. The results of the CFA are shown in Table 1.

**Table 1:** The results of the CFA of this study are shown in table.

Models	*χ* ^2^	Df	Δ*χ*^2^	RMSEA	CFI	TLI
M1: 4-factor model	539.03	489		0.02	0.99	0.99
M2: 3-factor model (combine cognitive conflict and affective conflict)	684.12	492	145.09***	0.04	0.95	0.95
M3: 3-factor model (combine shared leadership and cognitive conflict)	1035.34	492	496.31***	0.07	0.86	0.85
M4: 3-factor model (combine shared leadership and affective conflict)	893.97	492	354.92***	0.06	0.90	0.89
M5: 2-factor model (combine shared leadership, cognitive conflict and affective conflict)	1270.18	494	731.15***	0.08	0.80	0.79
M6: 1-factor model (combine shared leadership, cognitive conflict, affective conflict and team creativity)	1473.31	495	934.28***	0.09	0.75	0.74

Significance: ^+^*p* < 0.10; *p < 0.05; **p < 0.01; ***p < 0.001.

### 5.2 Descriptive statistics and correlations

Table 2 presents the descriptive statistics and zero-order correlations among the study variables. The mean scores for all variables were above the midpoint of the 5-point Likert scale, suggesting a generally positive perception of generational diversity, cognitive conflict, affective conflict, shared leadership, and team innovation performance in the sample. The standard deviations were relatively small, indicating limited variability in the responses.

**Table 2:** Descriptive statistics and zero-order correlations.

Variable	Mean	SD	1	2	3	4	5
1. Generational diversity	2.79	0.65	1				
2. Cognitive-based conflict	2.27	0.58	0.57***	1			
3. Affective-based conflict	2.30	0.56	0.50***	0.55***	1		
4. Shared leadership	3.47	0.69	0.01	0.23***	0.03	1	
5. Team creativity	3.43	0.71	0.15*	0.31***	−0.17*	0.25***	1

Significance: ^+^*p* < 0.10; *p < 0.05; **p < 0.01; ***p < 0.001.

The correlation analysis revealed several noteworthy associations. Generational diversity was significantly and positively correlated with both cognitive-based conflict (*r* = 0.57, *p* < 0.001) and affective-based conflict (*r* = 0.50, *p* < 0.001), supporting the notion that generational differences can trigger both task-related and relationship-related conflicts. Cognitive-based conflict was positively correlated with team innovation performance (*r* = 0.31, *p* < 0.001), suggesting that constructive disagreements can stimulate creativity and innovation. In contrast, affective-based conflict was negatively correlated with team innovation performance (*r* = −0.17, *p* < 0.05), highlighting the detrimental impact of interpersonal tensions on team outcomes. Shared leadership was positively correlated with cognitive-based conflict (*r* = 0.23, *p* < 0.001) and team innovation performance (*r* = 0.25, *p* < 0.001), but not significantly correlated with affective-based conflict (*r* = 0.03, *p* > 0.05).

These correlational findings provide preliminary support for our hypotheses and set the stage for further analysis using mediation and moderated mediation models.

### 5.3 Hypotheses test of mediation model

Before examining the mediation model, we first tested the direct effect of generational diversity on team creativity. The Mplus results did not indicate a significant effect of generational diversity on team creativity (*B* = 0.169, SE = 0.101, *p* = 0.093 > 0.05), hence hypothesis H1 was not supported.

To test the mediation model related to hypotheses H2 and H3, we examined the indirect effects of generational diversity on team innovation performance through cognitive-based conflict and affective-based conflict, respectively. The results revealed that generational diversity was significantly and positively related to both cognitive-based conflict (*B* = 0.530, SE = 0.059, *p* < 0.001) and affective-based conflict (*B* = 0.485, SE = 0.063, *p* < 0.001). Furthermore, cognitive-based conflict had a positive effect on team innovation performance (*B* = 0.647, SE = 0.086, *p* < 0.001), whereas affective-based conflict had a negative effect (*B* = −0.659, SE = 0.090, *p* < 0.001).

The indirect effects analysis showed that both cognitive-based conflict and affective-based conflict significantly mediated the relationship between generational diversity and team innovation performance. The indirect effect through cognitive-based conflict was positive and significant [indirect effect = 0.343, SE = 0.060, *p* < 0.001, 95% CI (0.239, 0.468)], supporting H2. The indirect effect through affective-based conflict was negative and significant [indirect effect = −0.320, SE = 0.064, *p* < 0.001, 95% CI (−0.463, −0.212)], supporting H3. These findings suggest that generational diversity can both enhance and hinder team innovation performance, depending on the type of conflict it triggers.

### 5.4 Hypotheses test of moderated mediation model

In the second model, we incorporated shared leadership as a moderator and examined its interaction effects with cognitive-based and affective-based conflicts on team innovation performance. The moderated mediation model demonstrated a good fit to the data [MLR *χ*^2^ (2) = 2.450, RMSEA = 0.080, SRMR = 0.018, CFI = 0.995, TLI = 0.901].

The analysis revealed a significant positive interaction effect between cognitive-based conflict and shared leadership in predicting team innovation performance (*B* = 0.617, SE = 0.113, *p* < 0.001). This finding suggests that the positive relationship between cognitive conflict and team innovation is amplified when shared leadership is high. In contrast, the interaction effect between affective-based conflict and shared leadership on team innovation performance was not significant (*B* = −0.087, SE = 0.134, *p* = 0.514), indicating that shared leadership does not moderate the negative relationship between affective conflict and team innovation. The unstandardized estimates of the model are presented in Table 3.

**Table 3:** Unstandardized estimates (standard error) of the moderated mediation path.

	Cognitive-based conflict	Affective-based conflict	Team innovation performance		
Control variable 1 Educational diversity	−0.03	−0.01	−0.06	−0.05	−0.03
Control variable 2 Tenure diversity	0.02	0.04	0.03	0.04	0.02
Independent variable generational diversity	0.53***	0.49***	0.17	0.17	0.09
Mediator 1 Cognitive-based conflict				0.65***	0.58***
Mediator 2 Affective-based conflict				−0.66***	−0.56***
Moderator Shared leadership					0.19**
Shared leadership*cognitive-based conflict					0.62***
Shared leadership*affective-based conflict					−0.09
*R* ^2^	0.33	0.26	0.03	0.28	0.30

Significance: ^+^*p* < 0.10; *p < 0.05; **p < 0.01; ***p < 0.001.

**Figure 2:**
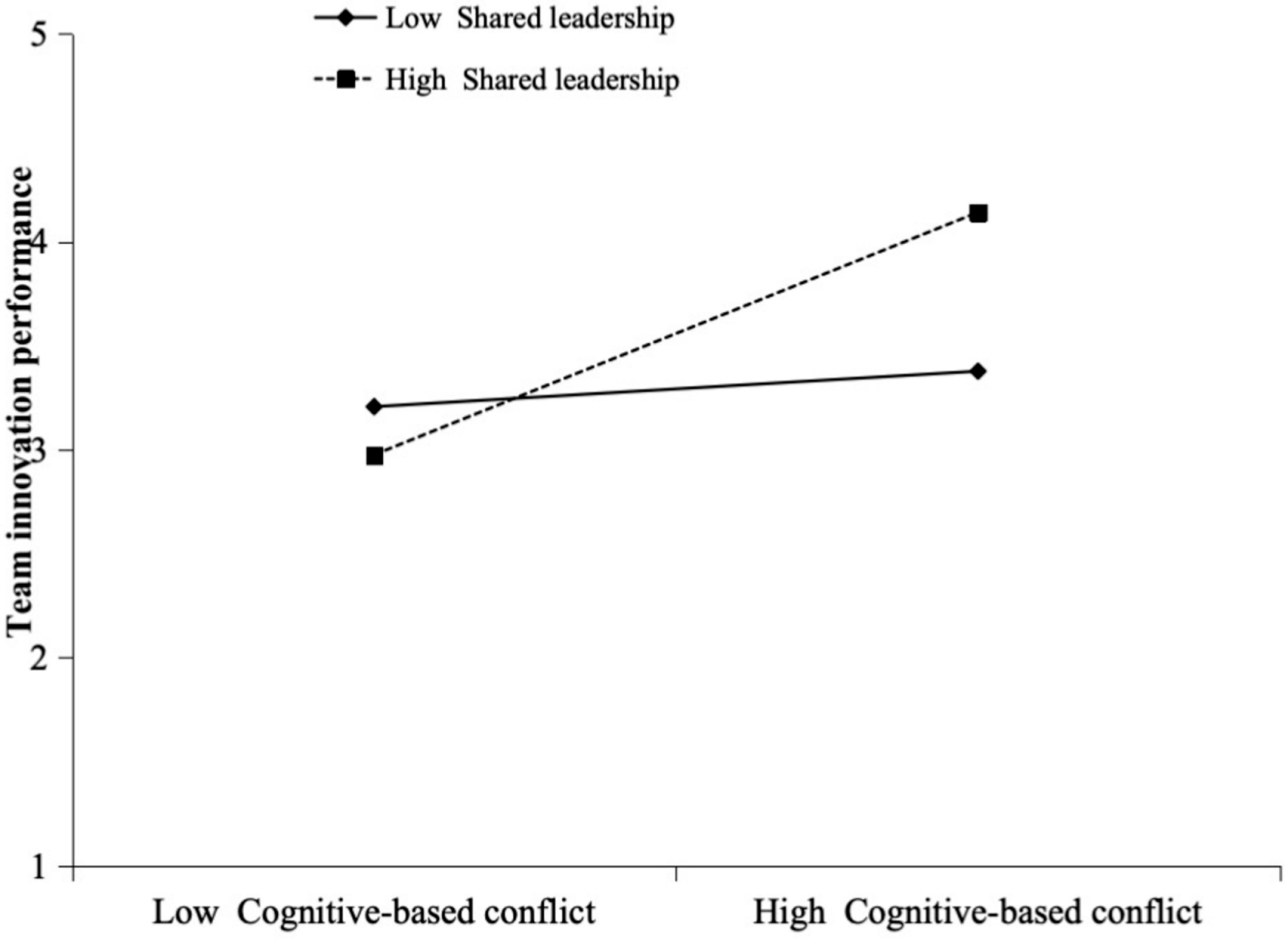
Interaction plot of cognitive-based conflict and affective-based conflict with shared leadership on team innovation performance.

Figure 2 illustrates the interaction plot using values plus and minus one standard deviation from the mean of shared leadership (Cohen et al., 2003).

The plot in Figure 2 shows that cognitive-based conflict positively influenced team innovation performance when shared leadership was high [*B* = 0.986, SE = 0.112, *p* < 0.001, 95% CI (0.774, 1.209)], but this relationship was not significant when shared leadership was low [*B* = 0.147, SE = 0.103, *p* = 0.153, 95% CI (−0.048, 0.357)], supporting H4. Conversely, affective-based conflict negatively influenced team creativity regardless of the level of shared leadership, whether high [*B* = −0.617, SE = 0.147, p < 0.001, 95% CI (−0.925, −0.346)] or low [B = −0.496, SE = 0.108, *p* < 0.001, 95% CI (−0.730, −0.306)], thus H5 was not supported.

We further analyzed the conditional indirect effect of generational diversity, as shown in Table 4.

**Table 4:** Comparison of moderated indirect effect.

Mediator	Pattern	Indirect effect	SE	*t*	*p*	95%CI
Cognitive-based conflict	Low shared leadership	0.08	0.06	1.38	0.17	−0.03, 0.20
	High shared leadership	0.53	0.08	6.37	0.00	0.38, 0.70
	Difference	0.45	0.10	4.73	0.00	0.27, 0.65
Affective-based conflict	Low shared leadership	−0.24	0.06	−3.85	0.00	−0.39, −0.14
	High shared leadership	−0.30	0.09	−3.54	0.00	−0.49, −0.16
	Difference	−0.06	0.09	−0.64	0.52	−0.26, 0.11

The indirect effect of generational diversity on team innovation performance via cognitive-based conflict was significantly stronger when shared leadership was high [indirect effect = 0.532, SE = 0.083, *p* < 0.001, 95% CI (0.378, 0.702)] compared to when shared leadership was low [indirect effect = 0.078, SE = 0.056, *p* = 0.167, 95% CI (−0.025, 0)].

The indirect effect through affective-based conflict was not significant, regardless of the level of shared leadership [indirect effect = −0.299, SE = 0.085, *p* = 0.000, 95% CI (−0.490, −0.160) for high shared leadership; indirect effect = −0.240, SE = 0.062, *p* = 0.000, 95% CI (−0.388, −0.140) for low shared leadership]. The difference in indirect effects was not significant [difference of indirect effect = −0.059, SE = 0.103, 95% CI (−0.285, 0.143)]. The results of the moderated mediation analysis supported H4a and H4b, but not H5a and H5b. The comparison of the moderated indirect effects is summarized in Table 4.

## 6 Discussion

Research on the correlation between work group diversity and group performance has produced inconclusive findings. Among the many aspects of diversity, generational diversity has emerged as a particularly contentious and debated subject. The existing literature on generational diversity and its impact on work outcomes presents a mixed picture. While some studies have reported limited effects of generational differences, particularly in specific fields like the medical sector (Parry and Urwin, 2011), others have highlighted the potential for both positive and negative outcomes depending on various contextual factors. The inconclusive nature of these findings underscores the need for more comprehensive investigations that delve deeper into the underlying mechanisms and processes through which generational diversity influences team dynamics and performance.

Recent research has also raised concerns about the methodological and theoretical limitations of existing studies on generational differences (Van Rossem, 2019). Many studies have relied on simplistic categorizations of generations and have failed to adequately account for the complex interplay of individual, team, and organizational factors that shape the impact of generational diversity. To address these limitations, our study adopts a more nuanced approach by integrating decision-making and social identity perspectives and incorporating key mediators and moderators.

By adopting this integrated theoretical framework, our research contributes to the existing literature in several ways. First, we provide compelling evidence that generational diversity has an indirect, rather than direct, impact on team innovation performance. This challenges the notion that generational differences alone are sufficient to explain variations in work-based outcomes (Costanza and Finkelstein, 2015). Second, we demonstrate that the indirect effect of generational diversity on team innovation is mediated by both cognitive and affective conflicts. This highlights the dual nature of generational diversity, which can simultaneously trigger both functional and dysfunctional conflicts within teams. Third, we examine the moderating role of shared leadership in this complex process, revealing its potential to amplify the positive effects of cognitive conflict and mitigate the negative effects of affective conflict.

Furthermore, our findings challenge the traditional view that shared leadership is universally effective in managing all types of conflict. While shared leadership appears to be effective in fostering an environment conducive to constructive debate and the leveraging of diverse perspectives, it may not be as effective in resolving interpersonal tensions and emotional conflicts. This suggests that different leadership styles or interventions may be required to address different types of conflict in multigenerational teams.

### 6.1 Theoretical implications

This study makes several significant theoretical contributions to the literature on generational diversity and team innovation. First, by integrating decision-making and social identity perspectives, we offer a more comprehensive and nuanced understanding of the mechanisms through which generational diversity influences team innovation performance. This integrated framework highlights the importance of considering both task-related and relationship-related factors in understanding the dynamics of multi-generational teams.

Second, our study challenges the prevailing notion that generational differences have a direct and deterministic impact on work-based outcomes. By demonstrating the mediating role of cognitive and affective conflicts, we provide evidence that the impact of generational diversity is contingent upon the types of conflict it triggers within teams.

Third, our findings underscore the importance of shared leadership in harnessing the potential benefits of generational diversity. By fostering an environment of open communication, collaboration, and constructive conflict resolution, shared leadership can amplify the positive effects of cognitive conflict on team innovation. However, our study also reveals the limitations of shared leadership in mitigating the negative effects of affective conflict, suggesting the need for further research on alternative leadership styles or interventions that may be more effective in addressing interpersonal tensions and emotional conflicts.

### 6.2 Practical implications

Our research offers valuable insights for managers seeking to effectively manage multigenerational teams and foster innovation in today’s diverse workplaces. First, our findings highlight the importance of recognizing the dual nature of generational diversity. While generational differences can lead to conflicts and challenges, they can also be a source of creativity and innovation. Managers need to be aware of both the potential benefits and drawbacks of generational diversity and adopt strategies to leverage the former while mitigating the latter.

Second, our study underscores the critical role of shared leadership in fostering team innovation in multi-generational contexts. By promoting a collaborative and inclusive leadership style, organizations can create an environment where diverse perspectives are valued and encouraged, and where cognitive conflict is channeled toward productive outcomes. However, managers also need to be mindful of the limitations of shared leadership in addressing affective conflict and consider implementing additional interventions, such as conflict resolution training or team-building activities, to address interpersonal tensions and emotional conflicts.

Third, our research suggests that effective management of multigenerational teams requires a nuanced and adaptive approach. Managers need to be attuned to the specific needs and challenges of different generations and tailor their leadership styles and interventions accordingly. By fostering an environment of mutual respect, understanding, and collaboration, organizations can harness the full potential of generational diversity and drive innovation in the workplace.

### 6.3 Limitation and future directions

Our study, while offering significant theoretical and practical insights, encounters certain limitations that warrant acknowledgment. Primarily, the reliance on self-reported data for most variables might introduce common method bias, a concern noted by Podsakoff et al. (2003). We attempted to mitigate this through a multi-wave design, gathering data at varied intervals. While self-reporting is appropriate for subjectively evaluated constructs like generational diversity and the mediating variables, future research should seek alternative data collection methods for validation.

The impact of team dynamics on innovation, influenced by internal factors like gender, discipline, and external elements such as incentive systems, is well-documented (Im et al., 2013). Our study controlled for demographic variables and work-related factors to isolate generational diversity’s unique effect. However, future studies should employ more robust methodologies, like longitudinal designs, to capture the evolving nature of generational diversity (Costanza et al., 2012).

Moreover, conflict management in teams might be affected by team atmosphere, norms, trust, and respect levels (Behfar et al., 2008; DeChurch et al., 2013; Jehn and Mannix, 2001). Future research should explore these factors as potential moderators in resolving conflicts in multigenerational teams. While our focus was on leadership style, subsequent research could broaden the scope to include these aspects, thus enhancing the comprehensiveness of the framework.

The implications of the scale adaptation and validation for the study’s findings, particularly in the context of Chinese financial firms, will be further explored in the Discussion section. We will also consider how these results might influence the generalizability of our findings and suggest directions for future research that could build upon our validation work. This includes the potential for cross-validation studies to enhance the robustness of the scales and their applicability in different cultural and organizational contexts.

### 6.4 Generational diversity and team innovation: a discussion on indirect effects

The findings of this study indicate that the direct effect of generational diversity on team innovation is not significant (*p* = 0.093), which may seem counterintuitive at first, given our emphasis on this relationship in the hypotheses. However, our analysis reveals that generational diversity significantly impacts team innovation indirectly through cognitive and affective conflicts. This discrepancy may arise from the complexity of generational diversity, which does not merely influence team innovation directly but shapes the cognitive and affective conflicts within the team, thereby indirectly affecting the innovation process.

Firstly, the multidimensional nature of generational diversity implies that employees from different generations may differ in values, work attitudes, and communication styles. These differences may not directly translate into innovative activities, but they do influence how team members communicate, collaborate, and resolve conflicts, all of which are critical factors in the innovation process. For instance, younger generations may be more inclined to adopt new technologies and innovative methods, while older generations may place more emphasis on tradition and experience. Such differences can lead to cognitive conflicts, which, if managed effectively, can foster innovative thinking and problem-solving.

Secondly, affective conflicts may stem from personal incompatibilities and emotional tensions, which are typically considered detrimental to team performance. However, our study demonstrates that with effective leadership and management, these conflicts can be transformed into a catalyst for team innovation. Thus, we emphasize the importance of understanding and managing these indirect effects, rather than focusing solely on the direct link between generational diversity and team innovation.

Lastly, these findings have significant implications for practitioners. They highlight the importance of nurturing open communication, collaboration, and conflict resolution skills within multi-generational teams, and how these can be leveraged to foster team innovation.

The limitations of our scales in cross-cultural research are an important consideration. While our methods have strived to ensure the scales’ applicability, there may still be nuances that are culturally specific and could affect the interpretation of the results. We suggest that future research continues to refine these scales by incorporating more diverse cultural perspectives and conducting comparative studies across a wider range of cultural groups. This will not only strengthen the validity of the scales but also enrich our understanding of how generational diversity influences team innovation in different cultural contexts.

To enhance the connection between our empirical findings and the theoretical frameworks of decision-making and social identity theories, we provide the following insights:

*Application of Decision-Making Theory*: Our empirical results offer valuable insights into how diversity within teams, particularly cognitive conflict, fosters deeper information processing and innovative thinking. This aligns with the decision-making theory’s proposition that diversity can enhance team performance by bringing a wider range of perspectives to the decision-making process.

*Application of Social Identity Theory*: We will analyze how our findings either support or challenge the social identity theory. Specifically, we will discuss how shared leadership mitigates the dynamics of in-group and out-group within multigenerational teams, thereby reducing affective conflict and enhancing team cohesion. This is directly related to the social identity theory’s assertion that group identity influences individual behaviors and intergroup relations.

*Alignment of Shared Leadership with Theories*: We will explore how our findings on shared leadership align with social identity theory. Our results indicate that shared leadership, by promoting mutual respect and trust among team members, helps to alleviate conflicts arising from generational differences. This is consistent with the view from social identity theory that harmony within a group can reduce intergroup conflicts.

*Explanation of Theories for Results*: We will further explain how these theories help us understand the differential moderating effects of shared leadership on cognitive and affective conflicts and how these conflicts impact team innovation performance.

By integrating these discussions, we aim to provide a more comprehensive understanding of how our empirical results are supported by and contribute to the existing theoretical frameworks. Given the insightful feedback on the cultural variability in generational classifications, we extend our discussion to address the cultural specificity of generational cohorts in China and its alignment with global patterns.

*Cultural Dependence of Generational Definitions*: Our categorization, while grounded in established literature, acknowledges the potential for variation in how generational cohorts are delineated across different cultures and societies. We will explore the nuances that may arise from differing cultural and societal contexts, affecting the universality and applicability of generational definitions.

*Comparison of Chinese Generational Experiences with Global Patterns*: We will contrast the generational experiences in China with global generational patterns to assess the degree of alignment. This involves examining how China’s unique socio-historical context shapes the values, work attitudes, and behavioral patterns of its generational cohorts, potentially deviating from global trends.

*Cultural Specificity Discussion*: We will delve into the cultural specificity of generational groups in China, analyzing how these specificities influence our research findings. This includes an in-depth look at the developmental backgrounds of Chinese generational cohorts and how these backgrounds inform their behaviors and interactions in the workplace.

*Implications for Research Findings*: Finally, we will discuss the potential impacts of these cultural specificities on our research outcomes and consider the applicability of our findings in other cultural contexts worldwide. This reflection aims to enhance the global relevance of our study and provide a more nuanced understanding of generational diversity’s role in team innovation performance across different cultures.

## 7 Conclusion

Our analysis demonstrates that generational diversity in teams leads to cognitive-based and affective-based conflicts, significantly influencing team innovation performance. We found that shared leadership plays a crucial role in moderating the cognitive process.

Our study, incorporating decision-making and social identity perspectives, reveals a dual process in managing team innovation performance in the context of generational diversity. While generational diversity can hinder team innovation performance through affective-based conflict, it can also enhance it via cognitive-based conflict. Importantly, shared leadership moderates cognitive process, intensifying the positive impact to yield beneficial work outcomes. Our analysis demonstrates that generational diversity in teams leads to cognitive-based and affective-based conflicts, significantly influencing team innovation performance. We found that shared leadership plays a crucial role in moderating the cognitive process. This aligns with the findings of Carson et al. (2007) and Wang et al. (2014), who also observed the mediating effects of leadership in managing generational diversity and enhancing team outcomes. This research contributes significantly to the understanding of generational diversity’s effects on team performance and offers valuable insights for managing multi-generational teams, especially from a leadership style standpoint.

Our research emphasizes the importance of understanding how generational diversity indirectly impacts team innovation through cognitive and affective conflicts. For managers, this means focusing not just on generational diversity itself but on the internal team dynamics as well. By fostering a work environment that promotes open communication, collaboration, and effective conflict resolution, organizations can better harness the potential of multi-generational teams to drive innovation.

## Data Availability

The original contributions presented in the study are included in the article/supplementary material, further inquiries can be directed to the corresponding author/s.
